# High Frequency of *PIK3R1* Alterations in Ovarian Cancers: Clinicopathological and Molecular Associations

**DOI:** 10.3390/cancers17020269

**Published:** 2025-01-15

**Authors:** Iwona K. Rzepecka, Andrzej Tysarowski, Bozena Konopka, Agnieszka Dansonka-Mieszkowska, Jolanta Kupryjanczyk

**Affiliations:** 1 Cancer Molecular and Genetic Diagnostics Department, Maria Sklodowska-Curie National Research Institute of Oncology, 02-781 Warsaw, Poland; andrzej.tysarowski@nio.gov.pl (A.T.); bozena.konopka@nio.gov.pl (B.K.); agnieszka.dansonka-mieszkowska@nio.gov.pl (A.D.-M.); 2 Department of Cancer Pathomorphology, Maria Sklodowska-Curie National Research Institute of Oncology, 02-781 Warsaw, Poland; jolanta.kupryjanczyk@nio.gov.pl

**Keywords:** *PIK3R1*, *PIK3CA*, PI3K, p85α, ovarian cancer, mutation, gene copy number, gene expression

## Abstract

Background: The phosphoinositide 3-kinase (PI3K) pathway is activated in multiple cancers. However, the significance of *PIK3R1* encoding the PI3K regulatory subunit, an inhibitor of the PI3K catalytic subunit encoded by *PIK3CA*, in ovarian cancer development is largely unknown. Methods: Here, we investigated *PIK3R1* genomic alterations and gene expression by direct sequencing and qPCR methods in 197 ovarian cancers. The results were correlated with clinicopathological and molecular variables and patient outcomes. Results: In addition to mutations (3.5%) and allelic losses (28.4%), we observed a very high frequency of decreased *PIK3R1* mRNA levels in ovarian carcinomas (95.8%). Tumors with *PIK3R1* mutations mostly represented low-stage cancers of endometrioid and clear-cell type. Tumors with *PIK3R1* deletion and underexpression shared similar phenotypes of high-grade carcinomas (*p* = 0.003 and *p* = 0.025, respectively). Allelic loss was also associated with advanced stages (*p* = 0.003) and high-grade serous histotypes (*p* = 0.004). The *PIK3R1* copy number correlated with mRNA levels (*p* = 0.009). *PIK3R1* mutations coexisted with *PTEN* mutations (*p* = 0.041), whereas *PIK3R1* deletion and underexpression were linked to *PIK3CA* amplification (*p* = 0.038 and *p* = 0.033, respectively). Low *PIK3R1* expression diminished the probability of a complete response (OR 0.07, *p* = 0.03) in patients treated with platinum-based regimens. Conclusions: *PIK3R1* alterations may contribute to the development of ovarian cancers with different malignant potential and molecular changes. The high frequency of *PIK3R1* aberrations suggests their importance in PI3K pathway deregulation, and they may potentially serve as an alternative to *PIK3CA* markers for therapy with these pathway inhibitors.

## Simple Summary

Phosphoinositide 3-kinase (PI3K) activation may occur via different mechanisms that involve defects in *PIK3R1* encoding the PI3K regulatory subunit (p85α). We aimed to evaluate *PIK3R1* alterations in ovarian cancers in the context of their clinicopathological characteristics, other molecular changes, and patient prognoses. *PIK3R1* aberrations were present in the majority of tumors with underexpression as the most frequent event. We found that *PIK3R1* mutations mainly contribute to the development of low-stage endometrioid and clear-cell carcinomas with coexisting *PTEN* mutations. In contrast, *PIK3R1* gene deletion and mRNA underexpression are features of aggressive high-grade cancers of different histological types with *PIK3CA* amplifications and *TP53* mutations. We also identified low *PIK3R1* mRNA expression as an unfavorable predictive factor for platinum-based therapy. Our data indicate the role of *PIK3R1* in ovarian cancer development and define a significant group of patients with different *PIK3R1* alterations that may potentially benefit from treatment with PI3K inhibitors.

## 1. Introduction

Ovarian cancer (OC) is the eighth leading cause of cancer death among women worldwide, with 324,398 new cases and 206,839 deaths in 2022 [1]. When it is diagnosed at the early stage of disease, the 5-year survival rate is very high (~90%). However, due to the lack of specific symptoms, many OCs are diagnosed in advanced stages, where the 5-year survival declines to ~30% [2].

Most OCs are of epithelial origin [3]. Histologically, epithelial OC is divided into five major subtypes: high-grade serous, endometrioid, clear-cell, mucinous, and low-grade serous [2]. Based on genetic alterations, disease development, and prognoses, epithelial OC can be further grouped into type I and type II carcinomas [4]. Type I tumors include slow-growing, low-grade serous, endometrioid, mucinous, and clear carcinomas characterized by mutations in *KRAS*, *PIK3CA*, *PTEN*, *BRAF*, *CTNNB1*, and *ARID1A*, mostly arising from endometriosis or borderline serous tumors. Type II tumors, which occur more frequently, are aggressive, high-grade serous carcinomas, carcinosarcomas, and undifferentiated carcinomas with a high prevalence of *TP53* and *BRCA1/2* mutations, distal fallopian tube origin, and poor prognoses compared to type I OC [3,5,6].

Despite the molecular diversity, most OCs are treated in the same manner, which includes radical surgery followed by carboplatinum and paclitaxel chemotherapy, with or without bevacizumab, an angiogenesis inhibitor. Depending on genetic test results on *BRCA1/2* mutations and homologous recombination deficiency (HRD), maintenance therapy may include poly (ADP-ribose) polymerase (PARP) inhibitors, bevacizumab, or a combination of both [7]. Unfortunately, despite achieving initial remission, up to 70% of patients will experience recurrence because of platinum resistance [8]. To improve therapeutic efficacy and patient survival, extensive research is underway to examine aberration in cancer signaling pathways, which would help to develop aberration-specific inhibitors.

Alterations in the PI3K pathway are common in cancers, making this pathway one of the most important for therapeutic intervention [9]. These alterations involve *PIK3CA* (p110α catalytic subunit of PI3K), *PIK3R1* (p85α regulatory subunit of PI3K), and *PTEN*, the negative regulator of PI3K. Upon activation, PI3K generates phosphatidylinositol 3,4,5-trisphosphate (PIP_3_), which activates critical downstream targets, such as AKT [10]. The induction of PI3K can be further enhanced by direct binding with activated RAS protein [11]. In physiological conditions, PI3K is mainly in the autoinhibited state. p85α inhibits and stabilizes the catalytic subunit, and it may also negatively regulate the PI3K pathway by forming homodimers that stabilize PTEN [12]. In addition to p85α’s canonical role in regulating the PI3K catalytic subunit, free p85α has a function in oxidative stress response, mediating apoptosis regulated by p53 [13,14].

Somatic mutations in the *PIK3R1* gene have been reported in 4.4% of all tumors, with the highest frequency in endometrial carcinoma (31%), glioblastoma multiforme (11%), and colorectal cancer (8%) [15,16]. The 5q13.1 region containing *PIK3R1* is frequently lost in breast (23%) and prostate (24%) cancers [17,18]. *PIK3R1* mRNA expression is also markedly lower in tumors than in normal tissues across multiple cancer types, including breast, lung, kidney, prostate, and endometrial carcinomas [17]. Low *PIK3R1* mRNA expression is associated with unfavorable prognoses in breast cancer patients, increased PI3K signaling, and the tumorigenic transformation of breast cancer models [17,18,19].

*PIK3R1* mutations are infrequent in ovarian cancers, except for the endometrioid and clear-cell types, in which they have been detected with a frequency ranging from 7% to 50% [20,21,22]. In contrast to mutations, *PIK3R1* copy number loss is frequently observed in high-grade serous ovarian cancers (HGSOCs) (68%) and is correlated with reduced *PIK3R1* mRNA expression [23]. *PIK3R1* loss favors ovarian tumorigenesis through the co-activation of AKT and STAT3 signaling and confers sensitivity to these pathway inhibitors [23].

The present study aimed to evaluate *PIK3R1* status in ovarian carcinomas by analyzing mutations, copy number alterations, and mRNA expression, in the context of the clinicopathological features of these tumors. We also related *PIK3R1* gene status to changes in other PI3K pathway components, like *PIK3CA* and *PTEN*, as well as to mutations in *TP53* and *KRAS*. Finally, we evaluated the clinical importance of *PIK3R1* alterations in ovarian cancer patients. Our data provide insight into the clinicopathological and molecular characteristics of tumors with *PIK3R1* dysfunction and may have important implications in the future planning of ovarian cancer therapy with PI3K pathway inhibitors.

## 2. Materials and Methods

### 2.1. Patients and Samples

The study group comprised 197 patients with ovarian carcinomas (age range: 17–88 years, median 53 years). The patients were treated in the Maria Sklodowska-Curie National Research Institute of Oncology between 1995 and 2009. The tumors were uniformly reviewed histopathologically and classified according to the World Health Organization (WHO) criteria [24]. There were 126 (64%) serous—including 109 (55%) high-grade serous ovarian cancers (HGSOCs) and 17 (9%) low-grade serous ovarian cancers (LGSOCs)—25 (13%) endometrioid, 19 (10%) clear-cell, 10 (5%) mucinous, 9 (5%) undifferentiated, and 8 (4%) mixed-type or unclassified carcinomas. Of the tumors, 12 (6%) were well differentiated (G1), 46 (23%) showed moderate differentiation (G2), and 139 (71%) were poorly differentiated (G3). Tumors were staged according to the criteria of the International Federation of Gynecologists and Obstetricians (FIGO) [25]. Of the tumors, 35 (18%) were in FIGO stage I, 10 (5%) in stage II, 140 (71%) in stage III, and 12 (6%) in stage IV.

*PIK3R1* mutations were analyzed in 141 cases including 74 serous, 25 endometrioid, 19 clear-cell, 10 mucinous, 7 mixed, and 6 undifferentiated ovarian cancers. The somatic copy number alteration (CNA) in the *PIK3R1* gene was analyzed for 197 ovarian cancers. *PIK3R1* mRNA expression analysis was carried out for 144 cases that met the criteria: at least 85% tumor cell content and sufficient quality of RNA.

Blood samples from healthy women were used to assess the normal copy number of the *PIK3R1* gene in CNA analysis. Five specimens of noncancerous fallopian tubes constituted a control group to compare the *PIK3R1* mRNA expression levels between normal and tumor tissues.

In a group of 151 patients treated with chemotherapy, 111 patients were cured with standard taxane–platinum (TP: paclitaxel with cisplatin or carboplatin), and 40 patients were cured with a platinum-based regimen (PC: cisplatin–cyclophosphamide or carboplatin–cyclophosphamide). Only patients with FIGO stage IIB-IV disease treated with standard protocols of chemotherapy were accepted for this study. The first-line chemotherapy consisted of 6 cycles in the TP-treated group and 6–8 cycles in the PC-treated group. Taxol was given in a 24 h (135 mg/m^2^) or 3 h infusion (175 mg/m^2^) and was followed by cisplatin (75 mg/m^2^) or carboplatin (AUC6). For the PC regimen, it was 75 mg of cisplatin/m^2^ or carboplatin (350 mg/m^2^ or AUC6) and 750 mg of cyclophosphamide/m^2^. Patients’ follow-up time ranged from 296 to 4062 days (median 1125 days) for the TP group and from 104 to 4080 days (median 887 days) for the PC group. Overall survival (OS) was defined as the interval from the date of initial surgical resection to the date of death or last contact. Disease-free survival (DFS) time was defined for patients who reached complete response (CR) as the interval from the date of the last chemotherapy course to the date of recurrence or last contact. Response to chemotherapy was evaluated retrospectively according to WHO response evaluation criteria based on data from medical records describing patient’s clinical condition and CA125 levels in 3–4 week intervals [26]. Clinical complete response (CR) was defined as the disappearance of all clinical and biochemical symptoms of ovarian cancers, evaluated after the completion of first-line chemotherapy and confirmed four weeks later. The platinum-sensitive group (PS) had a DFS time longer than six months.

### 2.2. DNA and RNA Extraction and cDNA Synthesis

Tumors and non-tumor tissues obtained during the surgical procedure (before chemotherapy) were snap-frozen in liquid nitrogen and stored at −68 °C. All tumor specimens were reviewed by a pathologist to ensure the maximal amount of tumor cells (mostly >70% for mutation and CNA analyses and at least 85% for mRNA analysis). Genomic DNA and total RNA were isolated using the QIAamp DNA Mini Kit (QIAGEN, Hilden, Germany) and the RNeasy Plus Mini Kit (QIAGEN), respectively. DNA and RNA quantity and quality were measured with a NanoDrop spectrophotometer (Thermo Fisher Scientific, Waltham, MA, USA), and additionally, RNA quality was assessed on an Agilent Bioanalyzer (Agilent Technologies, Santa Clara, CA, USA). One microgram of RNA was transcribed to cDNA using a High-Capacity cDNA Reverse Transcription Kit (Applied Biosystems, Vilnius, Lithuania) according to the manufacturer’s protocol.

### 2.3. PIK3R1 Mutation Analysis

The *PIK3R1* gene (GeneBank: NG_012849.2) consists of 16 exons with 15 exons encoding protein (from 2 to 16). All 15 protein-coding exons were searched for mutations with the use of Sanger sequencing. Primers were designed using Primer3web v4.1.0 (https://primer3.ut.ee) and checked for their specificity with Primer-BLAST (www.ncbi.nlm.nih.gov/tools/primer-blast, accessed on 20 November 2024) software. PCR mixtures were prepared according to the standard procedure (Applied Biosystems, Austin, TX, USA). Reactions were carried out on an Eppendorf thermocycler (Eppendorf, Hamburg, Germany) with an initial denaturation step at 95 °C for 5 min, followed by 36 cycles consisting of denaturation at 94 °C, annealing at 60 °C, and extension at 72 °C, each for 30 s. PCR products were further purified with exonuclease I and alkaline phosphatase (EURx Ltd., Gdansk, Poland) treatment and sequenced with a BigDye Terminator v3.1 Cycle Sequencing Kit (Applied Biosystems, Austin, TX, USA) on a 3500 Genetic Analyzer (Applied Biosystems).

### 2.4. PIK3R1 Copy Number Quantification

The *PIK3R1* somatic copy number alteration (CNA) was assessed with quantitative real-time PCR (qPCR) using TaqMan Copy Number Assays (Applied Biosystems) on a 7500 Fast Real-Time PCR system (Applied Biosystems). Reactions were carried out as a duplex in a 10 µL mixture containing 10 ng genomic DNA, TaqMan Universal Master Mix II, primers, and probes for *PIK3R1* (TaqMan Copy Number Assay Hs06008293_cn) and for the reference gene (TaqMan Copy Number Reference Assay RNase P). The conditions of the qPCR were as follows: 95 °C for 10 min, 40 cycles at 95 °C for 15 s, and 60 °C for 1 min. Samples were analyzed in four replicates. Each 96-well plate included a negative control without a DNA template (NTC) and a calibrator sample with a known copy number for the target of interest. The number of copies of the *PIK3R1* sequence was determined by relative quantitation using the comparative C_T_ (ΔΔC_T_) method and CopyCaller Software v2.0 (Applied Biosystems). This method measures the C_T_ difference (ΔC_T_) between the target and reference sequence and then compares the ΔC_T_ values of the test samples to a calibrator sample known to have two copies of the target sequence. The copy number of the target (Q) is calculated to be two times the relative quantity. Copy number gain and loss were assigned as Q > 2.5 and Q < 1.5, respectively.

### 2.5. PIK3R1 mRNA Expression Quantification

*PIK3R1* expression at the mRNA level was evaluated with the use of the quantitative real-time PCR (qPCR) method and TaqMan Gene Expression Assays (Applied Biosystems) on a 7500 Fast Real-Time PCR system. qPCRs were run as separate reactions for the target and each of the reference genes, *HPRT1*, *PPIA,* and *GUSB*. Reactions were carried out in three replicates in a 10 µL mixture containing 11 ng cDNA, TaqMan Universal Master Mix II with UNG, and the TaqMan Gene Expression Assay for *PIK3R1* (Hs00933163_m1) or for the reference gene (*HPRT1*, 4326321E; *PPIA*, 4326316E; or *GUSB*, 4326320E). Each 96-well plate included negative controls (NTC) for the analyzed genes. The thermal profile of the qPCR was as follows: 50 °C for 2 min (Uracil-N-Glycosylase activity), 95 °C for 10 min, followed by 40 cycles consisting of two steps: 95 °C for 15 s and 60 °C for 1 min. The comparative C_T_ (ΔΔC_T_) method was used to analyze changes in the *PIK3R1* expression normalized to the mean of the reference gene expression in a given sample relative to a calibrator sample. The sample exhibiting the highest expression of the *PIK3R1* gene was used as a calibrator. *PIK3R1* expression was analyzed as a categorical variable with the median value of the expression used as a cut-off point.

### 2.6. Statistical Analysis

Comparison between groups was performed using the Mann–Whitney U test for continuous variables and the Chi-squared or Fisher’s exact test for categorical variables. Analyses of prognoses were performed using the univariate and multivariate Cox proportional hazards models and further confirmed with the log-rank test and Kaplan–Meier survival estimates. The univariate and multivariate logistic regression models were used to analyze predictions. In all multivariate models, the *PIK3R1* alterations were correlated with clinicopathological tumor characteristics, including patient age (categorized by median split), histological type (categorization: HGSOCs vs. other types), grade (categorization: grade III vs. I-II), clinical stage (FIGO IIIC-IV vs. I-IIIB), residual tumor size (categorization: Rt < 2 cm vs. 0 and Rt ≥ 2 cm vs. 0), and type of chemotherapy regimen (TP vs. PC). All multivariate statistical models were simplified by backward stepwise elimination of variables if their *p*-values were higher or equal to 0.1. The level of significance was set as *p* < 0.05. All calculations were performed using STATA v11 software.

## 3. Results

### 3.1. PIK3R1 Mutations

**Figure 1:**
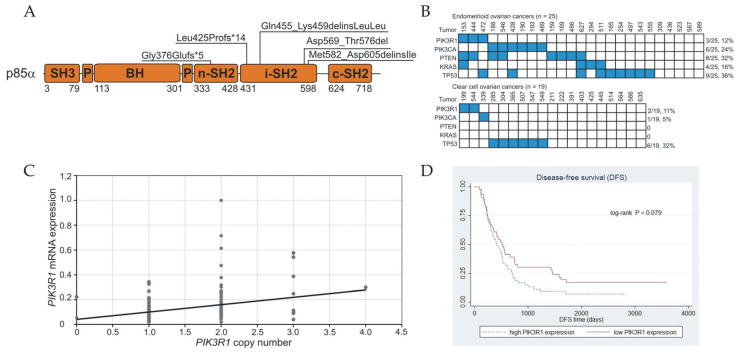
*PIK3R1* alterations in ovarian cancers: (**A**) the location of individual mutations within the *PIK3R1* gene in ovarian cancers. (**B**) *PIK3R1*, *PIK3CA*, *PTEN*, *KRAS*, and *TP53* mutations in endometrioid and clear-cell ovarian cancers. Mutated tumors (blue) are distinguished from tumors with no detectable mutation (white). The mutation frequency for individual genes is shown on the right. (**C**) *PIK3R1* gene copy number plotted against *PIK3R1* mRNA expression normalized to reference genes. The tumor (case 337) with the highest expression (equaling 1) was used as the calibrator. The line is shown to visualize trends in the expression change. (**D**) Prognostic significance of *PIK3R1* expression. Patients with low *PIK3R1* mRNA levels in ovarian tumors tended to have better disease-free survival (DFS) than patients with high expression (Kaplan–Meier curve).

Five *PIK3R1* mutations were identified in 141 (3.5%) ovarian carcinomas (Table 1, Figure 1A). All mutations were deletion or deletion–insertion type and were localized within the nSH2 and iSH2 domains. Two truncating mutations were found in exon 9, two in-frame changes were detected in exons 10 and 12, and one deletion was in the intron 12 splice acceptor site. cDNA sequencing revealed that the intronic mutation leads to the deletion of 69 bp encoded by exon 13 and probably results in the exon 13 skipping during transcription and the insertion of a single residue Ile.

Among tumors harboring *PIK3R1* mutations, we observed the loss of the *PIK3R1* allele in one tumor, amplification in two tumors, and the retainment of both alleles in another two tumors. In the case of amplification, we did not determine which allele, with or without mutation, was amplified.

Mutations were detected in three endometrioid (12%, 3/25), two clear-cell (10.5%, 2/19), and none of the 74 serous or 10 mucinous cancer cases. Most tumors with mutations were FIGO I or II, moderately differentiated carcinomas. Women with *PIK3R1*-mutated and -unmutated tumors had a mean age at diagnosis of 58.4 and 54.3 years, respectively.

We compared *PIK3R1* mutations with *PIK3CA*, *PTEN*, *KRAS,* and *TP53* status previously determined in these tumors [27,28,29]. *PIK3R1* mutations were mutually exclusive with *PIK3CA* mutations. They were associated with *PTEN* mutations (*p* = 0.041) but not with *KRAS* mutations and tended to coexist with *TP53* wild-type tumors (*p* = 0.076, Table 2). In detail, two endometrioid tumors with a *PIK3R1* mutation had a coexisting *PTEN* mutation, and one of them also had a mutation in the *KRAS* gene. Another endometrioid tumor harbored a concurrent mutation in *TP53*. In contrast, in clear-cell cancers, *PIK3R1* mutations occurred alone.

The prevalence of PI3K pathway mutations, including *PIK3R1*, *PIK3CA*, and *PTEN* was 13.5% (19/141) in ovarian cancers. In the endometrioid histological type, this frequency was 52% (13/25, Figure 1B) and was significantly higher than in clear-cell type cancers (15.8%, 3/19, *p* = 0.025), LGSOCs (6.7%, 1/15, *p* = 0.005), and HGSOCs (1.7%, 1/59, *p* < 0.001). Because *KRAS* mutations can stimulate PI3K activity and were observed in 16% (4/25) of endometrioid tumors, the frequency of the PI3K pathway aberration due to *PIK3R1*, *PIK3CA*, *PTEN*, and *KRAS* mutations may increase to 60% (15/25) in this histotype.

### 3.2. PIK3R1 Copy Number Alterations

The number of *PIK3R1* copies ranged from 0.25 to 3.7 (mean value 1.93). The copy number alteration was observed in 70 of 197 (35.5%) ovarian cancers. Specifically, there were 28.4% (56/197) allelic losses and 7.1% (14/197) amplifications at the *PIK3R1* locus.

The gene allele loss was detected in HGSOCs (36.7%, 40/109) and LGSOCs (11.8%, 2/17) and endometrioid (16%, 4/25), clear-cell (26.3%, 5/19), mixed-type (25%, 2/8), mucinous (10%, 1/10), and undifferentiated (22.2%, 2/9) ovarian carcinomas. The allelic loss was more common in HGSOCs than in other tumors (36.7%, 40/109 vs. 18.2%, 16/88, *p* = 0.004, Table 3). This is in contrast to *PIK3R1* mutations, which were observed exclusively in endometrioid and clear-cell types. Moreover, *PIK3R1* copy loss was associated with adverse clinicopathological features. The loss of *PIK3R1* was more frequently observed in advanced FIGO IIIC-IV stages (34.8%, 47/135) than in FIGO stage I-IIIB cancers (14.5%, 9/62, *p* = 0.003). Similarly, high-grade cancers had a higher frequency of allelic losses (34.5%, 48/139) than low-grade carcinomas (13.8%, 8/58, *p* = 0.003). There was no relationship between allele loss and patient age.

We investigated the potential relationship between *PIK3R1* and *PIK3CA*, *PTEN*, *KRAS*, or *TP53* alterations described in our previous studies [27,28,29]. *PIK3R1* copy loss was associated with both *PIK3CA* amplification (*p* = 0.038) and *PTEN* allele loss (*p* = 0.006, Table 2). Interestingly, there seemed to be an association between *PIK3R1* copy loss and decreased or absent PTEN protein expression. The frequency of tumors that showed *PIK3R1* copy loss steadily increased from tumors with strong PTEN protein expression (14%, 6/41), to those with moderate expression (38%, 14/37), to those with decreased or absent PTEN protein expression (41%, 14/34, *p* = 0.019). The loss of the *PIK3R1* allele was more common in *TP53*-mutated cancers than in *TP53* wild-type tumors (35.8%, 48/134 vs. 12.7%, 8/63, *p* = 0.001). *PIK3CA*, *PTEN,* and *KRAS* mutations were not associated with *PIK3R1* allele loss.

*PIK3R1* amplification was found in HGSOC (8.3%, 9/109), LGSOC (5.9%, 1/17), endometrioid (8%, 2/25), and clear-cell (10.5%, 2/19) histological types. There were no significant associations between this alteration and clinicopathological or molecular factors, except for *PIK3R1* mutations. Tumors with *PIK3R1* amplifications harbored *PIK3R1* mutations more frequently than tumors without amplification (20%, 2/10 vs. 2.3%, 3/131, *p* = 0.040).

### 3.3. PIK3R1 mRNA Expression

*PIK3R1* mRNA expression was significantly decreased in ovarian cancers compared with control tissues (*p* = 0.003, Mann–Whitney U test). The difference in mean expressions between these groups was 69% (0.116 ± 0.11 and 0.376 ± 0.31 in ovarian cancers and control tissues, respectively). *PIK3R1* mRNA was downregulated in 95.8% (138/144) of ovarian cancers relative to the control group’s mean, indicating the relevance of *PIK3R1* in ovarian tumorigenesis.

We examined *PIK3R1* expression in relation to DNA copy number loss. The samples were divided into those with low and high *PIK3R1* mRNA levels according to the median expression in the cancer group (0.0989) (n = 72 in each group). Low expression may be a consequence of allelic loss of the *PIK3R1* gene because there is an association between mRNA level and the copy number alteration of the gene (*p* = 0.009, Figure 1C). DNA copy number loss was more frequently observed in tumors with low mRNA expression (45.8%, 33/72) than in tumors with high expression (25%, 18/72, *p* = 0.009). There was no significant relationship between *PIK3R1* mutations and mRNA levels.

We assessed *PIK3R1* expression levels in ovarian cancer subtypes. Low expression was present in HGSOCs (49.5%, 50/101) and endometrioid (75%, 6/8), clear-cell (83%, 5/6), mixed-type (71%, 5/7), and undifferentiated (75%, 6/8) cancers but not in LGSOCs (0%, 0/13) or mucinous (0%, 0/1) ovarian cancers. The difference in the expression levels between HGSOCs and LGSOCs was statistically significant (*p* <0.001). Interestingly, diminished expression was more common in the endometrioid and clear-cell types than in serous type (75.6%, 11/14 vs. 43.9%, 50/114, *p* = 0.021).

Next, we examined *PIK3R1* expression in relation to clinicopathological and molecular factors. Lower levels of *PIK3R1* mRNA were more common in poorly differentiated than in well and moderately differentiated tumors (54%, 65/120 vs. 29%, 7/24, *p* = 0.025). There was no relationship between expression and clinical stage or patients’ age (Table 3). We found an association between expression and *PIK3CA* amplification or *TP53* mutations. Low *PIK3R1* expression was more frequent in cancers with *PIK3CA* amplifications than those with *PIK3CA* WT tumors (64%, 14/22 vs. 38%, 24/64, *p* = 0.033). Similarly, tumors with diminished *PIK3R1* expression tended to harbor *TP53* mutations more often than *TP53* WT tumors (53%, 62/116 vs. 36%, 10/28, *p* = 0.092, Table 3). There was no association between *PIK3R1* expression and *PTEN* or *KRAS* changes (Table 2).

### 3.4. PIK3R1 Alterations as Prognostic and Predictive Factors

Univariate and multivariate statistical analyses were performed to assess the associations of *PIK3R1* copy number alteration and expression with patients’ outcomes in the entire group of patients and subgroups treated with either the standard taxane–platinum (TP) or the platinum-based (PC) regimen. In the group analyzed for mRNA expression, 103 patients were treated with TP regimens and 34 with PC.

Low *PIK3R1* expression diminished the probability of complete response (CR) in the PC-treated patients. Low expression was rarely observed in patients who reached CR (29%, 7/24) compared to those with other responses (70%, 7/10, *p* = 0.028). In both univariate and multivariate analyses, low expression negatively influenced the probability of CR (OR 0.18, *p* = 0.035 and OR 0.07, *p* = 0.030, respectively, Table 4). There were no associations between *PIK3R1* expression and CR in the TP-treated and the entire group of patients. *PIK3R1* expression was not associated with platinum sensitivity in any of the analyzed groups.

Low *PIK3R1* expression tended to diminish the risk of recurrence in the entire group of patients. The mean disease-free survival (DFS) time of patients with low and high expression was 569 and 470 days, respectively. Univariate and multivariate analyses showed a trend toward better prognoses for patients with low *PIK3R1* expression compared to those with high expression in terms of recurrence (HR = 0.68, *p* = 0.081, log-rank, *p* = 0.079, and HR = 0.064, *p* = 0.054, respectively; Figure 1D). DFS also showed associations with grade and debulking status. These correlations were not confirmed in the smaller TP and PC-treated patient subgroups. Patients’ overall survival was not influenced by the *PIK3R1* mRNA level in either group.

There were no associations between clinical endpoints and the *PIK3R1* DNA copy number.

## 4. Discussion

The current study showed that the vast majority of ovarian cancers carry *PIK3R1* alterations (>90%). We identified mutations, gene deletions, and a very high frequency of mRNA underexpression. We also found that tumors with *PIK3R1* mutations had other clinicopathological and molecular characteristics than tumors with gene loss or reduced mRNA expression.

*PIK3R1* mutations are rare in ovarian cancer. In our study, this alteration was observed in 3.5% of tumors, which is similar to the rate (0–3.8%) reported by other groups [15,21,30,31]. All mutations were clustered within the iSH2 and nSH2 domains involved in PI3K inhibition and stabilization. It has been shown that mutations in these domains disrupt the inhibitory interface and retain the stabilizing interconnection between the regulatory and catalytic subunits, resulting in PI3K activation [15,32]. In contrast, p85α with truncating mutations N-terminal to iSH2 cannot bind the p110α subunit and probably acts independently of PI3K via the activation of JNK signaling [33,34]. In our series, two truncating mutations were identified, which may represent this PI3K-independent course of action. The distribution of mutations we observed was similar to that found in endometrial cancers, in which the vast majority of mutations (93.3%) clustered within the iSH2 and nSH2 domains [35]. In contrast, in prostate cancers, the truncating mutations were found mainly in the cSH2 domain, suggesting that the localization of *PIK3R1* mutations may vary between tumor types and may have different functional consequences [17].

We found mutations mostly in low-stage, moderately differentiated endometrioid carcinomas (12%) and in clear-cell (10.5%) carcinomas, which is in agreement with the scarce literature on this subject. To date, Cybulska et al. has reported mutations in 11.1% of analyzed endometrioid ovarian cancers, mostly low-stage, and in 0.5% of HGSOCs from The Cancer Genome Atlas [20]. Fieuws detected *PIK3R1* mutations in 6.7% of clear-cell carcinomas and 2.4% of HGSOCs with no clinical characteristics given [21]. Teer et al. found *PIK3R1* mutations in 50% of endometrioid ovarian cancers [22].

This study revealed *PIK3R1* copy number alterations, including allelic losses (28.4%) and amplifications (7.1%). We also observed that these allele losses were associated with an advanced FIGO stage, high tumor grade, and HGSOC histotype. The association between the loss of a gene allele and clinicopathological parameters of ovarian cancers has not been previously reported. The chromosome region with *PIK3R1* was frequently deleted in HGSOCs according to TCGA data [36]. Similarly, Huang et al. reported significantly frequent copy deletions in the *PIK3R1* region in serous histotypes [37]. A detailed analysis of the *PIK3R1* copy number in serous ovarian cancer across TCGA revealed 68.4% heterozygous and 3.5% homozygous loss, respectively [23]. However, a small fraction of *PIK3R1* allele amplification (3.3%) was also observed in TCGA ovarian cancer patients [23]. Moreover, Teer et al. found one endometrioid ovarian cancer with both *PIK3R1* mutation and amplification [22]. Investigations performed on other cancer types demonstrated *PIK3R1* copy number loss in 23% of breast cancers, as well as in 24% and 36% of primary and metastatic prostate cancers, respectively [17,18]. Data from murine models revealed that, for both *Pik3r1* and *Pten*, heterozygous mice display increased AKT activity and increased intestine neoplasia compared with *Pten* heterozygotes alone [38]. In addition, single-copy ablation of *Pik3r1* accelerated a mouse model of HER2-/neu-driven breast cancers [18]. Collectively, these data suggest that *PIK3R1* copy number losses are frequent in ovarian cancers and other cancer types and may have tumorigenic potential. However, the clinicopathological characteristics and clinical significance of this alteration has not yet been fully explored.

Our study revealed that *PIK3R1* is lowly expressed in ovarian cancers. Similarly, low *PIK3R1* mRNA expression was found in ovarian cancer and many other human cancers in the OncoMine microarray database [39]. Decreased expression of *PIK3R1* was also observed in serous ovarian cancers in TCGA cohorts [23,40] and, in agreement with our results, was correlated with the gene copy numbers, suggesting that reduced expression may be related to gene allele loss [23]. However, opposite results of *PIK3R1* and p85α overexpression have also been reported in ovarian tumors [41]. In our study, we found diminished mRNA expression in all the histological types analyzed. Still, it was significantly more frequent in endometrioid and clear-cell types than in serous types and was inversely associated with the grade of malignancy. Low *PIK3R1* expression has previously been linked to advanced histological grade and/or clinical stage in other carcinomas, like in breast cancer [19], neuroblastoma [42], hepatocellular cancer [39], and others [17,43].

p85α can inhibit PI3K activity not only by binding and inhibiting the catalytic subunit but also by competing with PI3K to bind to receptors. This is because p85 is in excess of the catalytic subunit in cells [44]. Thus, the binding of p85 monomers to receptors prevents interactions between the p85-p110 complex and RTKs, resulting in reduced PI3K signaling. This mechanism of PI3K inhibition is particularly sensitive to free p85α levels. In fact, decreased *PIK3R1* expression mainly affects free p85 monomers, resulting in a significant increase in PI3K signaling. Indeed, even transient knockdown of *PIK3R1* (leading to a partial loss of *PIK3R1* mRNA expression) alone was sufficient to induce AKT activation and the proliferation of prostate cancer cells [17]. Additionally, it was shown that serous ovarian cancer cell lines with *PIK3R1* loss demonstrated multiple tumorigenic properties like increased proliferation, migration, and invasion [23]. Consequently, in mice, reduced *PIK3R1* expression promoted oncogenic transformation and metastatic dissemination of ovarian cancer [23]. Together, these data support the function of *PIK3R1* as a tumor suppressor, the loss of which may enhance tumorigenesis in ovarian cancer and other cancer types.

Regarding the changes in other genes of the PI3K pathway, we found that *PIK3R1* mutations are mutually exclusive with *PIK3CA* and coexist with *PTEN* mutations. This is in agreement with a study of a pan-cancer cohort across 20 different cancer types, including ovarian cancer, in which only 2 patients out of the 1200 analyzed harbored both a *PIK3R1* and a *PIK3CA* mutation [45]. Similarly, a study on breast cancers did not reveal the coexistence of mutations in the regulatory and catalytic PI3K subunits [19]. However, *PIK3R1* and *PIK3CA* mutations co-occur in endometrial [35] and colorectal cancers [15], suggesting that these double events may contribute to pathogenesis in particular tumor types.

Several studies have shown that *PIK3R1* frequently coexists with *PTEN* and *KRAS* mutations in endometrial tumors [35,46]. According to Cheung and colleagues, *PIK3R1* mutations may co-occur with heterozygous *PTEN* mutations to compensate for the incomplete loss of PTEN protein [46]. It was revealed that the sole *PIK3R1* mutations present in normal uterine endometrium are not sufficient to initiate malignant transformation. Still, they are associated with a higher tendency towards endometriosis development, while endometriotic epithelia carrying additional mutations in *PTEN* are more likely to transform into endometriosis-associated ovarian cancer [47]. These data, together with our findings, indicate that mutations in *PIK3R1* and *PTEN* or *KRAS* may work together for efficient transformation into endometrioid ovarian cancer that may represent type I tumors in ovarian cancer classification.

In contrast to *PIK3R1* mutations, *PIK3R1* allele loss and decreased mRNA expression are associated with aggressive clinicopathological features and unfavorable molecular changes, indicating that they may contribute to type II tumor development. These molecular alterations include *PIK3CA* amplification, *PTEN* loss, and *TP53* mutations. Similar to low *PIK3R1* expression, *PIK3CA* amplification can disrupt the levels of free p85α leading to PI3K pathway activation. This is because an excess of p110α, due to *PIK3CA* amplification, can sequester a surplus of free p85α monomers into the p85α-p110α complex, increasing the amount of PI3K enzymes [46]. Moreover, an excess of p110α may disturb free p85α homodimerization. p85α homodimers are able to bind and stabilize PTEN protein, protecting PTEN from WWP2-mediated proteasomal degradation [13]. Thus, apart from competing with PI3K to bind to RTKs, p110α-free p85α can negatively regulate the PI3K pathway through PTEN stabilization, whose function may be attenuated by *PIK3CA* amplification.

*TP53* is the most frequently mutated gene in HGSOCs [16]. In our series consisting of different histological types, *TP53* mutations were present in the majority of tumors. TP53 activates the transcription of numerous genes directing cell cycles, DNA repair, or apoptosis in response to DNA damage [48]. The role of free p85α in cellular stress pathways has also been confirmed [13]. In response to oxidative stress, apoptotic cell death mediated by p53 was blocked in cells with little to no p85α expression [14,49]. Moreover, free p85α is a positive regulator of p53 acetylation and a critical upstream proapoptotic mediator in the UV-B response [50]. In addition, p85α is upregulated by p53 at the transcription level, further indicating proapoptotic roles for p85α in response to cellular damage [13,14].

In this study, we observed a decreased probability of complete response in PC-treated patients with low *PIK3R1* mRNA expression. Our finding is consistent with the proapoptotic function of free p85α via the positive regulation of p53, which may be weakened in cells with low p85α amounts. Similarly, a study on serous ovarian cancer cell lines demonstrated that *PIK3R1* downregulation induced the expression of antiapoptotic *BCL2*, as well as genes involved in cell cycle progression (*CCNB1*) and metastasis (*MMP9* and *VEGFA*) [23]. However, an opposite effect of enhanced apoptotic response to platinum treatment in platinum-resistant cells was also observed in HGSOC cell lines with *PIK3R1* knockdown [51]. Nevertheless, our findings imply that *PIK3R1* loss and *TP53* mutations may have a negative, additive influence on the response to platinum-based therapy. On the other hand, low *PIK3R1* expression tended to diminish the risk of recurrence in the entire group of patients. This observation is surprising, since in our study low expression was associated with other pathological factors linked to poor outcomes, like high nuclear grade. We propose that the decreased risk of recurrence may be attributed to a significantly higher proportion of endometrioid and clear-cell types than HGSOCs in the group with low expression. These two histological types of ovarian cancer develop more slowly and are usually diagnosed at a lower clinical stage than HGSOCs even with comparable high nuclear grades. Contrary to this conclusion, an ovarian serous cystadenocarcinoma analysis from TCGA revealed an association between high *PIK3R1* expression and worse prognoses [40]. This discrepancy in these study results indicate that the prognostic significance of *PIK3R1* expression warrants further investigations in ovarian cancer patients. Nevertheless, it should be underlined that low *PIK3R1* expression was a negative prognostic factor in multiple cancer types, including prostate, gastric, liver, and non-small-cell lung cancers as well as breast and uterine endometrial carcinomas [17,40].

Alterations in the PI3K pathway found in a wide variety of human cancers provided the rationale for the development of PI3K/AKT inhibitors, and some of them have been clinically approved [9]. Unfortunately, no PI3K-pathway-targeted drugs have been approved for ovarian cancer treatment so far. Several preclinical studies suggest the possibility that PI3K pathway inhibitors might be effective in *PIK3R1*-deficient tumors. D’Ambrosio et al. demonstrated that an ovarian cancer patient-derived xenograft (PDX) with the *PIK3R1* mutation W624R was sensitive to the pan-class I PI3K inhibitor buparlisib, and also to the p110α specific inhibitor alpelisib, as well as to the dual PI3K and mTOR inhibitors dactolisib [52]. In another study, *PIK3R1* copy number loss or reduced *PIK3R1* expression rendered ovarian cancer cells vulnerable to the inhibition of AKT or JAK2/STAT3 inhibitors [23]. In a mice model of breast cancer, pan-PI3K and p110α-selective pharmacological inhibition effectively blocks transformation driven by partial p85α loss [18]. These data suggest that PI3K pathway therapeutics may be effective in the treatment of ovarian cancer patients with *PIK3R1* loss. Recently, Passarelli et al. reported the outcomes of patients with *PIK3CA*-mutated recurrent gynecological tumors, including 10 ovarian cancers, prospectively treated with alpelisib within a controlled program (NCT04085653) [53]. In the ovarian cancer group, the disease control rate (DCR) was 50% (4: stable disease; 1: partial response), and the median progression-free survival (PFS) was 5.0 months. Our results and those of preclinical studies suggest that *PIK3R1* alterations like *PIK3R1* gene deletion—common in type II cancers—may serve as an alternative to *PIK3CA* markers for therapy with these pathway inhibitors and should be taken into consideration while planning future clinical trials.

## 5. Conclusions

In summary, we showed that the *PIK3R1* gene is frequently altered in ovarian cancers due to mutations, DNA copy number alterations, and decreased *PIK3R1* mRNA expression. We found that *PIK3R1* mutations mainly contribute to the development of low-stage endometrioid and clear-cell carcinomas with coexisting *PTEN* mutations. In contrast, copy number loss and decreased mRNA expression are features of aggressive high-grade ovarian cancers representing different histological types harboring *PIK3CA* amplifications and *TP53* mutations. Moreover, we found that low *PIK3R1* expression significantly diminished the probability of complete response in patients treated with platinum-based regimens. To our knowledge, this is the first ovarian cancer study comprising the analysis of *PIK3R1* mutations, copy number alterations, mRNA expression with respect to clinicopathological and molecular factors, and patients’ endpoints. This study may be important since we found that there is a significant group of ovarian cancer patients with different *PIK3R1* alterations that may potentially benefit from treatment with PI3K and AKT inhibitors.

## Figures and Tables

**Table 1:** *PIK3R1* mutations in ovarian cancers.

Case No.	Age (Years)	Histology ^1^	Grade	FIGO	Exon/Intron	Nucleotide Change	Predicted Effect on Protein	*PIK3R1* Copy Number	Scc ^2^ (%)
199	50	C	3	IC	exon 9	c.1128delG	p.(Gly376Glufs*5)	2.87	10
472	88	E	3	IIB	exon 9	c.1274_1289delinsCTGTTGGT	p.(Leu425Profs*14)	1.50	5
153	45	E/MMCa	2	IA	exon 10	c.1364_1376delinsTTTT	p.(Gln455_Lys459delinsLeuLeu)	2.23	20
444	58	E/MMCa	2	IIIC	exon 12	c.1705_1728del	p.(Asp569_Thr576del)	3.04	10
544	51	C	2	IA	intron 12	c.1746-2_1746-4delTCA	p.(Met582_Asp605delinsIle)	2.22	<30

^1^ C—clear-cell, E—endometrioid, E/MMCa—endometrioid and mucinous mullerian carcinoma. ^2^ Scc—stromal cell contamination.

**Table 2:** *PIK3R1* mutations, copy number alterations, and mRNA expression in relation to molecular factors in ovarian cancers.

Variable	*PIK3R1* Mutations (N = 141)				*PIK3R1* Copy Number (N = 197)				*PIK3R1* mRNA Expression (N = 144)			
	N	Mutated (%)	WT (%)	*p*	N	Allelic Loss (%)	≥2 Alleles (%)	*p*	N	<Median (%)	>Median (%)	*p*
*PIK3CA*												
Mutation	7	0 (0)	7 (5)		7	0 (0)	7 (6)		0	-	-	-
WT	134	5 (100)	129 (95)	1	150	43 (100)	107 (94)	0.191	104			
*PIK3CA*												
Amplification	20	0 (0)	20 (21)		26	12 (35)	14 (18)		22	14 (37)	8 (17)	
Two alleles	78	2 (100)	76 (79)	1	88	22 (65)	66 (82)	**0.038**	64	24 (63)	40 (83)	**0.033**
*PTEN*												
Mutation	10	2 (40)	8 (6)		11	2 (5)	9 (8)		4	1 (2)	3 (5)	
WT	131	3 (60)	128 (94)	**0.041**	151	40 (95)	111 (92)	0.730	105	52 (98)	53 (95)	0.619
*PTEN*												
LOH	34	1 (33)	33 (31)		39	18 (50)	21 (24)		32	16 (39)	16 (38)	
Two alleles	74	2 (67)	72 (69)	1	83	18 (50)	65 (76)	**0.006**	53	24 (61)	29 (62)	0.673
*KRAS*												
Mutation	11	1 (20)	10 (7)		11	1 (2)	10 (8)		4	1 (4)	3 (4)	
WT	130	4 (80)	126 (93)	0.338	154	41 (98)	113 (92)	0.293	108	53 (96)	55 (96)	0.619
*TP53*												
Mutation	86	1 (20)	85 (63)		134	48 (86)	86 (61)		116	62 (86)	54 (75)	
WT	55	4 (80)	51 (38)	0.076	63	8 (14)	55 (39)	**0.001**	28	10 (14)	18 (25)	0.092

N—number of cases. WT—wild type. Significant changes are shown in bold.

**Table 3:** *PIK3R1* mutations, copy number alterations, and mRNA expression in relation to clinicopathological factors in ovarian cancers.

Variable	*PIK3R1* Mutations (N = 141)				*PIK3R1* Copy Number (N = 197)				*PIK3R1* mRNA Expression (N = 144)			
	N	Mutated (%)	WT (%)	*p*	N	Allelic Loss (%)	≥2 Alleles (%)	*p*	N	<Median (%)	>Median (%)	*p*
Age (years)												
<54	67	3 (60)	64 (47)		100	26 (46)	74 (52)		71	39 (54)	32 (44)	
≥54	74	2 (40)	72 (53)	0.668	97	30 (54)	67 (48)	0.443	73	33 (46)	40 (56)	0.243
Histology												
HGSOCs	59	0	59 (43)		109	40 (71)	69 (49)		101	50 (69)	51 (71)	
Others	82	5 (100)	77 (57)	0.075	88	16 (29)	72 (51)	**0.004**	43	22 (31)	21 (29)	0.856
FIGO stage												
I–IIIB	54	4 (80)	50 (37)		62	9 (16)	53 (38)		23	11 (15)	12 (17)	
IIIC-IV	87	1 (20)	86 (63)	0.071	135	47 (84)	88 (62)	**0.003**	121	61 (85)	60 (83)	0.82
Grading												
G1, G2	56	3 (60)	53 (39)		58	8 (14)	50 (35)		24	7 (10)	17 (24)	
G3	85	2 (40)	83 (61)	0.386	139	48 (86)	91 (65)	**0.003**	120	65 (90)	55 (76)	**0.025**

N—number of cases. WT—wild type. Significant changes are shown in bold.

**Table 4:** Probability of complete response (CR) and relative risk of recurrence (DFS) in the multivariate logistic regression and Cox’s proportional hazards model analyses obtained for the PC-treated and the whole group of ovarian carcinomas.

Variable	Complete Response (CR) PC-Treated Group, N = 34			Disease-Free Survival (DFS) All Patients (PC + TP), N = 98		
	HR/OR	95% CI	*p*	HR/OR	95% CI	*p*
*PIK3R1* expression						
Low vs. high	0.07	[0.01–0.78]	**0.030**	0.64	[0.41–1.01]	0.054
Age (years)						
≥54 vs. <54	0.08	[0.01–1.01]	0.051	-	-	-
Residual tumor						
Rt < 2 cm vs. 0 cm	-	-	-	1.71	[1.09–2.70]	**0.021**
Grading						
G III vs. I-II	-	-	-	2.27	[1.20–4.28	**0.011**

N—number of patients. Significant changes are shown in bold.

## Data Availability

The data presented in this study are available on request from the corresponding author.
